# Characterizing the Palm Pathogenic *Thielaviopsis* Species from Florida

**DOI:** 10.3390/jof10040247

**Published:** 2024-03-26

**Authors:** Marie-Gabrielle Ayika, Avril Rosano, Jacqueline Valiente, Seemanti Chakrabarti, Jeffrey A. Rollins, Braham Dhillon

**Affiliations:** 1 Department of Plant Pathology, Fort Lauderdale Research and Education Center, Institute of Food and Agricultural Sciences, University of Florida, Davie, FL 33314, USA; ayikam@ufl.edu (M.-G.A.); seemanti@ufl.edu (S.C.); 2 Institute of Food and Agricultural Sciences, College of Agricultural and Life Sciences, University of Florida, Gainesville, FL 32611, USA; rosanoavril@ufl.edu; 3 Miami Dade College, Miami, FL 33132, USA; jvaliente@archbold-station.org; 4 Department of Plant Pathology, Institute of Food and Agricultural Sciences, University of Florida, Gainesville, FL 32611, USA; rollinsj@ufl.edu

**Keywords:** *Thielaviopsis ethacetica*, cryptic species, fungicide, mating type, high-temperature stress, wounding, pathogenicity

## Abstract

*Thielaviopsis paradoxa* sensu lato is a soilborne fungal pathogen that causes Thielaviopsis trunk rot and heart rot in palms. The loss of structural integrity resulting from trunk rot can cause the palm trunk to collapse suddenly and poses a serious threat to life and property. Even though rudimentary knowledge about the *Thielaviopsis* infection process in palms is available, nothing is known about the *T. paradoxa* species complex in the US. The aim of this study was to characterize *T. paradoxa* s. lat. isolates collected from diseased palms grown in Florida. Multi-locus phylogeny using three genes, ITS, β-tubulin, and tef1-α, revealed that the isolates separate into two distinct clades with high bootstrap support. The majority of the isolates clustered with the species *T. ethacetica*, while two isolates formed a separate clade, distinct from *T. musarum*, and might represent an undescribed *Thielaviopsis* species. One representative isolate from each clade, when grown on three distinct media and at four different temperatures, showed differences in gross colony morphology, as well as growth rates. The *T. ethacetica* isolate TP5448 and the *Thielaviopsis* sp. isolate PLM300 grew better at opposite ends of the temperature spectrum tested in this study, i.e., 35 °C and 10 °C, respectively. In pathogenicity assays on whole plants, the *T. ethacetica* isolate proved to be more aggressive than *Thielaviopsis* sp. isolate PLM300, as it produced larger lesions when inoculated on wounded leaflets. An unequal distribution was observed for the mating-type locus of *T. ethacetica*, as 12 isolates carried the MAT1-1-1 allele, while the status for four isolates remained undefined. Variation in mycelial growth in response to different fungicides was also observed between the two clades. These results demonstrate the existence of two *Thielaviopsis* clades that can infect palms in Florida and underscore the need for targeted sampling to help uncover the diversity of *Thielaviopsis* species across palm-growing regions in the US.

## 1. Introduction

*Thielaviopsis paradoxa* sensu lato (s. lat.) is a fungal pathogen responsible for causing trunk and heart rot in palms. It is a soil-borne pathogen that usually enters through wounds and can colonize all palm tissues including the roots, trunk, leaves, inflorescence, and fruit [1]. In trunk rot, usually, no outward symptoms of decline are visible on *Thielaviopsis*-infected palms, either in the canopy or on the trunk [2]. Decay caused by *Thielaviopsis* only becomes apparent when the palm trunk folds over and collapses or the crown snaps off and falls to the ground [3]. The loss of structural integrity due to *Thielaviopsis* trunk rot can pose a serious risk to human life and property, especially when palms are planted for landscape aesthetics in high-traffic areas along sidewalks, parking lots, and in backyards.

*Thielaviopsis paradoxa* s. lat. was first described by De Seynes in 1886 on pineapple and named as *Sporochisma paradoxum*. It was labeled as *Chalara paradoxa* in 1892 by Saccardo and later identified on sugarcane in 1893 by Went as *Thielaviopsis ethaceticus*. In 1904, von Höhnel introduced the term *Thielaviopsis paradoxa* and recognized that it was synonymous to *Sprochisma paradoxum* De Seynes and *Thielaviopsis ethaceticus* Went. Subsequent work ultimately led to the recognition of *T. paradoxa* s. lat. as a discrete genus, *Thielaviopsis*, within the family Ceratocystidaceae, with constituent species having similar morphological and ecological traits [4]. Using molecular phylogeny supplemented by morphological comparisons, six species were delineated among *T. paradoxa* s. lat. isolates collected in Cameroon (Africa) and other culture collections sourced from around the world, including *T. paradoxa* s. str., *T. radicicola*, *T. musarum*, *T. euricoi*, *T. cerberus*, and *T. ethacetica* [5]. The latter was found to be the most cosmopolitan and affecting the broadest range of plants. Controlled pairing of different isolates established that *T. ethacetica* has a heterothallic mating system [5].

*Thielaviopsis* species are characterized by long neck ascomata with a bulbous base that produces sticky masses of ascospores [6] and two types of asexual spores, endoconidia and aleurioconidia. Primary endoconidia are hyaline, aseptate, cylindrical, and produced in phialides, whereas secondary endoconidia are barrel-shaped and start out as hyaline and melanize as they mature [7,8]. Aleurioconidia, or chlamydospores, are thick-walled resting spores that are produced singly or in chains at the ends of specialized conidiophores [9,10,11].

*Thielaviopsis* species are primarily pathogens of monocots like pineapple, sugarcane, banana, and palms. Palms (family Arecaceae) are perennial monocots with 2600 species grouped in 181 genera [12]. Palms are an integral feature of the landscape in tropical regions around the world, and commodities from palms, like dates, coconuts, and palm oil, form an important part of the global economy and trade. In Florida and other states in the subtropical US, palms are largely grown as ornamental in the landscape industry, with palms accounting for 10% (USD 404 M) of the plant sales by nurseries and greenhouses in Florida [13].

*Thielaviopsis* trunk rot was first detected on palms growing in Florida when *T. paradoxa* s. lat. was isolated from a diseased coconut palm [14]. Histology studies on diseased tissues obtained from sugarcane and date palm trunks show that fungal mycelia primarily invade the parenchyma cells, leaving the vascular bundles intact [1,15]. Even though rudimentary knowledge about the *Thielaviopsis* infection process in palms is available, nothing is known about the *T. paradoxa* species complex in the US. The aim of this study was to characterize the *T. paradoxa* s. lat. isolates collected from diseased palms grown in Florida for pathogenicity, growth, and response to different fungicides.

## 2. Materials and Methods

### 2.1. Fungal Isolates and Growth Conditions

Seventeen isolates from three palm species growing in Florida, collected over a period of 17 years (2005–2022), were used in this study (Table 1). Briefly, diseased palm trunk was split open, and 6–7 black, infected, 5 cm long fibers from the trunk were plated on half-strength potato dextrose agar (PDA; BD DIFCO Cat # 90000-758) media. The fungal growth on Petri plates was examined, and isolates with melanized spores arranged in chains, analogous to *Thielaviopsis* endoconidia, were selected, transferred to fresh Petri plates, and single-spored. These isolates were grown on PDA media at 28 °C in the dark. Agar plugs from the margins of actively growing fungal cultures were used for propagation.

### 2.2. DNA Extraction, PCR, and Sequencing

Fungal tissue was collected from two-day-old actively growing cultures, and the protocol for the rapid mini preparation of fungal DNA [16] was used with slight modifications to extract DNA. Briefly, mycelia were scraped from 1 cm^2^ of the agar surface and added to 1 ml of lysis buffer (400 mM Tris-HCl [pH 8.0], 60 mM EDTA [pH 8.0], 150 mM NaCl, 1% sodium dodecyl sulfate) in a 1.5 mL microcentrifuge tube with two 3 mm glass beads. A FastPrep-24™ Classic bead beating grinder (MP Biomedical Cat # 6004500) was used for two cycles of 4.5 m/s (130 rpm) for 30 s to disrupt the mycelia. The microcentrifuge tubes were incubated at room temperature for 10 min; 250 μL of potassium acetate (pH 4.8) was added and vortexed for 10 s. The supernatant was collected after centrifugation at 12,000× *g* for 2 min and transferred to a new tube. An equal volume of ice-cold isopropyl alcohol was added and mixed by inversion. The tube was centrifuged again at 12,000× *g* for 5 min and the supernatant was discarded. The DNA pellet was washed with 500 μL of 70% alcohol and air-dried for 10 min. The pellet was finally dissolved in 50 μL of sterile water and the DNA was used for PCR.

Three barcoding genes, nuclear ribosomal DNA internal transcribed spacer (nrDNA ITS), β-tubulin (β-tub), and translation elongation factor 1-α (tef1-α), were amplified from the 17 isolates, as previously described [5]. Primer pairs ITS1/ITS4 [17] and bt1a/bt1b [18] were used for ITS and β-tub, respectively. The tef1-α was amplified using two primer pairs, EF1F/EF2R [19] and EF1-526F/EF1-1567R [20]. The primer sequences and PCR cycling conditions are summarized in Table 2. The amplified DNA fragments were shipped to Eurofins Genomics, Louisville, KY, for sequencing.

### 2.3. Phylogeny

The Sanger sequences from the forward and reverse primers were used to generate a consensus sequence for the ITS, β-tub, and tef1-α amplicons from each isolate. The sequences for the three genes generated in this study were deposited in GenBank (Table 1). These sequences were aligned to three published NCBI PopSet datasets from 32 *Thielaviopsis* isolates, accession numbers 513044848 (ITS), 513044880 (β-tub), and 513044784 (tef1-α) [5], using MAFFT v7.310 [22] with default parameters in Jalview [23], and the alignment was curated manually. The species *Davidsoniella virescens* was used as the outgroup [5]. The multiple sequence alignment for the three genes was concatenated using FASconCAT-G [24]. A maximum likelihood phylogeny was generated using the PhyML 3.0 [25] web interface that utilized smart model selection (SMS) [26] to determine a substitution model based on the Bayesian information criterion (BIC). Branch support was calculated using the Shimodaira–Hasegawa-like approximate likelihood-ratio test (SH-like aLRT) method available in PhyML, and the phylogeny was visualized in FigTree v1.4.4 [27].

### 2.4. Morphology, Growth, and Thermotolerance

Two isolates, TP5448 and PLM300, representative of the two clades identified in this study, were grown on three solid media, potato dextrose agar (PDA; BD DIFCO Cat # 90000-758), Czapek Dox agar (CZD) [28], and minimal media (MM) [28], at 28 °C in the dark, and colony morphology was observed at 3 d. Growth of the two isolates was also monitored at five temperatures, 10, 20, 28, 35, and 40 °C, in complete dark. As the *Thielaviopsis* isolates are routinely grown in the lab at 28 °C in the dark, two temperatures lower and higher than 28 °C were selected to measure mycelial growth. Experiments for monitoring colony morphology and growth at different temperatures had a minimum of two replicates, and the experiment was repeated twice.

### 2.5. Microscopy

For microscopic observations, fungal tissues were excised with agar from PDA cultures and mounted on a microscope slide with a coverslip in deionized water. Observations were made with a Leica DM R compound light microscope (Leica Microsystems GmbH, Wetzlar, Germany) using differential interference contrast optics. Photographs of fungal structures were captured with a mounted Leica-DFC450 CCD digital camera (Leica Microsystems GmbH, Wetzlar, Germany) operated through the Leica Application Suite X software (Version 2.0.0 Build 14332; Leica Microsystems Ltd. Heerbrugg, Switzerland).

### 2.6. Determination of Mating Type

The mating-type (MAT) locus was examined in 17 *Thielaviopsis* isolates using PCR and mating assays. The complete nucleotide sequence and primers to amplify the *T. ethacetica* MAT1-1-1 allele were available [21]. The MAT1-2-1 primers were designed from conserved regions identified by aligning MAT1-2-1 sequences, MF476807 and BK010318, from two species, *T. paradoxa* and *T. punctulata*, respectively [21].

The mating assay, as described earlier [5], was carried out on 0.5× PDA plates that had thin slivers (0.5 cm thick and 8 cm long) of autoclaved petiole tissue from a silver thatch palm (*Cocothrinax argentata*) placed in the middle. All isolates were co-inoculated with isolate TP5448 (*T. ethacetica*) for at least 2 weeks in the dark at 28 °C, and the development of fruiting bodies was monitored in the interaction zone where the two isolates converged on the autoclaved petiole tissue. The experiment was repeated four times.

### 2.7. Pathogenicity Tests

Two isolates, TP5448 (*T. ethacetica*) and PLM300 (*Thielaviopsis* sp.), were used to inoculate whole plants and fruits. The whole-plant inoculation was carried out using five-year-old *Coccothrinax guantanamensis* potted palms. An agar plug carrying the fungal mycelium with conidia was placed on an incision wound made on the leaflet and wrapped with parafilm to maintain the moisture. The inoculated and non-inoculated wound sites were examined 3 days post inoculation (dpi) for lesion development. The lesion area was calculated by using a modified formula for the surface area of an ellipse, S = π (L × W)/4, where L is the lesion length and W is the width of the lesion in mm. Koch’s postulates were established by surface-sterilizing the inoculated and non-inoculated leaflets and plating on half-strength PDA. Additionally, mature fruits from Costa Rica bamboo palm (*Chamaedorea costaricana*) were surface sterilized and inoculated with isolates PLM300 and TP5448 using a spore suspension at a concentration of 1 × 10^6^ spores/mL.

### 2.8. Fungicide Sensitivity

The two isolates, TP5448 and PLM300, were grown on 0.2× PDA media supplemented with fungicides at 22 °C in the dark. Agar plugs from the margins of actively growing fungal cultures were used for propagation. A total of ten fungicides, AGphite 57, Banner Maxx II, Concert II, Headway G, Heritage WG, Medallion WDG, Mural WG, PHOSPHO-jet, Postiva, and RES505 (Table 3), were used at four concentrations, 0.1, 1, 10, and 100 μg ml^−1^, to test their efficacy in limiting the growth of the two isolates. Each isolate was grown in duplicate, and the experiment was repeated twice. The colony diameter was measured, and percent growth inhibition (PGI) was calculated as (1 − (growth on treatment/growth on control)) × 100. The EC50 value, the fungicide dose that inhibits fungal growth by 50% in in vitro assays, was calculated by fitting the relative growth values against the log concentration using a four-parameter log-logistic model (LL.4) using the ec50estimator package [29] in R, version 4.1.1 (2021-08-10)—“Kick Things”. Fungicides that showed less than 50% growth inhibition at the highest dose tested were excluded.

## 3. Results

A total of 17 *Thielaviopsis* isolates, collected from the trunks of diseased palms showing rot symptoms, were characterized for pathogenicity, phylogenetic relationships, and several other biological traits. All isolates were collected from three palm species growing in the state of Florida over the course of 17 years (Table 1).

### 3.1. Phylogenetic Relationships

**Figure 1:**
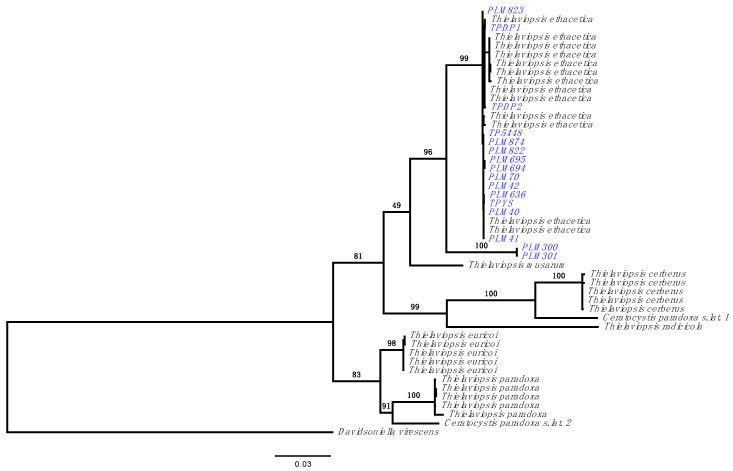
Phylogenetic relationship based on maximum likelihood (ML) analysis using three-gene (ITS, β-tubulin, and tef1-α) combined dataset. Sequences derived from *Thielaviopsis paradoxa* s. lat. isolates collected from diseased palms in Florida (highlighted in blue) were aligned to three published NCBI PopSet datasets from 32 *Thielaviopsis* isolates, accession numbers 513044848 (ITS), 513044880 (β-tub), and 513044784 (tef1-α) [5]. The branch labels represent percent bootstrap support. *Davidsoniella virescens* was used as an outgroup.

Sequence data for three fungal barcoding regions, nuclear ribosomal DNA internal transcribed spacer (nrDNA ITS), β-tubulin (β-tub), and translation elongation factor 1-α (tef1-α), were generated and used to determine the phylogenetic relationship among 16 isolates (Table 2) relative to previously published *Thielaviopsis* species [5]. The tef1-α region could not be amplified for isolate PLM873 (Table 1) so it was excluded from the phylogenetic analysis. The maximum likelihood (ML) phylogeny split the isolates into two groups, with 14 isolates grouped with the *T. ethacetica* clade and two isolates, PLM300 and PLM301, that formed a distinct clade separate from *T. ethacetica* and *T. musarum*, with strong bootstrap support (Figure 1). Thus, these two isolates appear to constitute a cryptic species that has not been described or named yet and will be referred to as *Thielaviopsis* sp. henceforth. The tef1-α region from the two isolates PLM300 and PLM301 was found to be 100% identical to the sequence from isolate ‘C 1481’, an undescribed *Thielaviopsis* species collected from a *Phoenix* palm [10]. Single-gene phylogeny using the tef1-α region grouped the three isolates, PLM300, PLM301, and C1481, into the same clade, separate from other described *Thielaviopsis* species (Figure S1), supporting the existence of cryptic species of *Thielaviopsis* in FL.

One representative isolate from the two groups, *T. ethacetica* TP5448 and *Thielaviopsis* sp. PLM300, was selected for further analysis.

### 3.2. Growth and Colony Morphology

**Figure 2:**
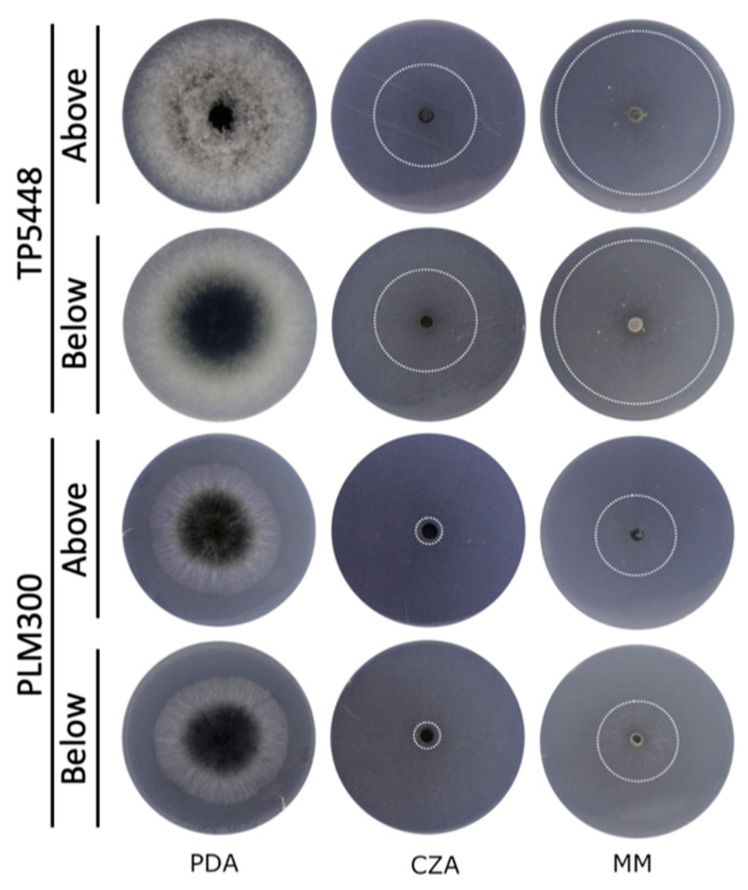
Colony morphology of *Thielaviopsis ethacetica* isolate TP5448 and *Thielaviopsis* sp. isolate PLM300 on three solid culture media. Colony characteristics for the two isolates were observed from the top and bottom of the Petri plates with potato dextrose agar (PDA), Czapek Dox agar (CZA), and minimal media (MM).

**Figure 3:**
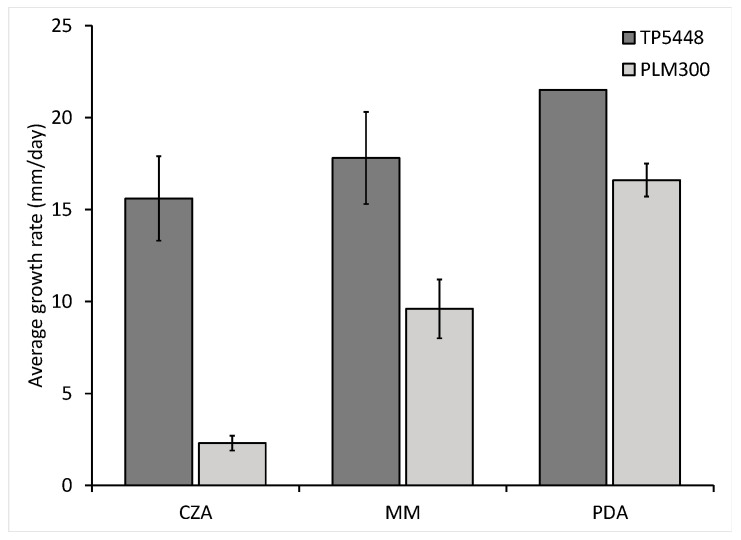
Growth rate of *Thielaviopsis ethacetica* isolate TP5448 and *Thielaviopsis* sp. isolate PLM300 on three solid culture media. Mycelial growth of cultures growing on Czapek Dox agar (CZA), minimal media (MM), and potato dextrose agar (PDA) was measured.

The growth of *Thielaviopsis* isolates from the two groups was measured on three media, namely potato dextrose agar (PDA), Czapek Dox agar (CZA), and minimal media (MM). The *T. ethacetica* and *Thielaviopsis* sp. isolates displayed a similar growth pattern on all three media. Both isolates exhibited very sparse growth on MM and CZA, spreading in a string-like fashion that was hard to observe macroscopically (Figure 2), whereas robust growth was observed on PDA (Figure 3).

On PDA, both isolates grew radially and presented a melanized zone in older growth, while pigmentation was lacking in the young mycelia that appeared white. The colony margins for *Thielaviopsis* sp. isolate PLM300 were very organized and well defined, while margins for isolate TP5448 had an irregular appearance when viewed from the underside (Figure 2). The top view for *T. ethacetica* isolate TP5448 showed fluffy cottony growth across the entire colony, while isolate PLM300 had a flat appearance. These traits were found to be consistent among the other members of the same clade. The distinct differences in colony morphology for *T. ethacetica* isolate TP5448 and *Thielaviopsis* sp. isolate PLM300 seen on PDA suggest that PDA was a suitable media for morphologically distinguishing the two clades (Figure 2).

**Figure 4:**
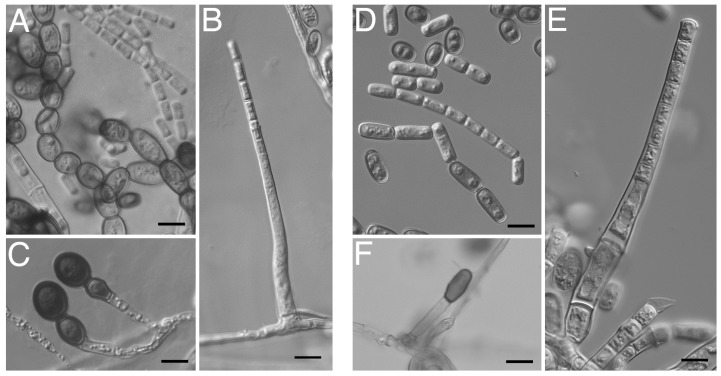
Spore morphology of *Thielaviopsis ethacetica* isolate TP5448 and *Thielaviopsis* sp. isolate PLM300. Chains of endoconidia (**A**) TP5448 and (**D**) PLM300. Primary endoconidia oozing out of phialidic conidiophores (**B**) TP5448 and (**E**) PLM300. Thick-walled aleurioconidia (**C**) TP5448 and (**F**) PLM300. Scale bar = 10 μm.

The two spore types, endoconidia and aleurioconidia, associated with *Thielaviopsis* were observed in both isolates (Figure 4). The characteristic chains of cylindrical and barrel-shaped endoconidia were observed extensively in two-day-old cultures (Figure 4A,D), along with cylindrical endoconidia exuding out of the external walls of the conidiophores (Figure 4B,E). The thick-walled aleurioconidia were also formed in chains (Figure 4C).

### 3.3. Thermotolerance

**Figure 5:**
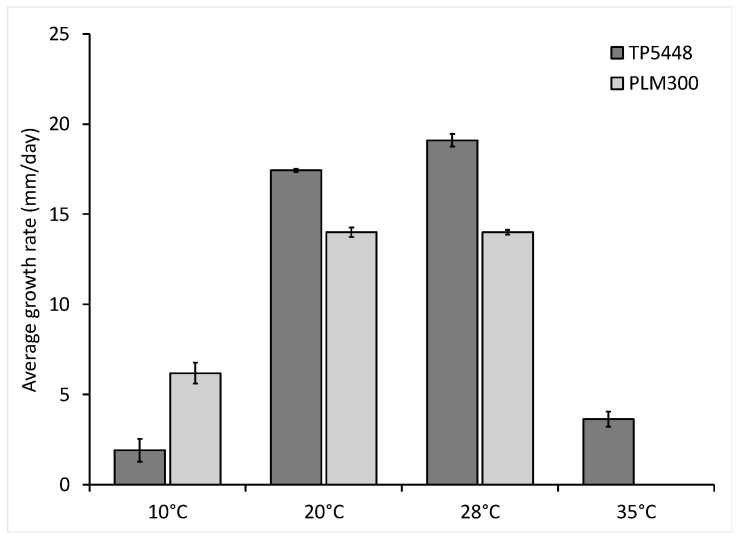
Growth rate of *Thielaviopsis ethacetica* isolate TP5448 and *Thielaviopsis* sp. isolate PLM300 at different temperatures. Optimal temperature for growth for both isolates was 28 °C; however, both isolates performed differently at the two temperature extremes, with TP5448 growing at higher temperature (35 °C) and PLM300 showing better growth at low temperature (10 °C).

The growth response of the two *Thielaviopsis* species at five temperatures, 10, 20, 28, 35, and 40 °C, was also measured, and a clear difference in growth rate was observed (Figure 5). The highest growth rate for both isolates was seen at 28 °C, whereas both species failed to grow at 40 °C, the highest temperature that was tested. In general, as the temperature warmed up from 10 °C to 28 °C, an increase in growth was observed for both isolates. However, the two isolates preferred to grow at the opposite ends of the temperature spectrum that was tested. Higher growth for *Thielaviopsis* sp. isolate PLM300 was seen at the cooler temperature, 10 °C (Figure 5). On the other hand, the *T. ethacetica* isolate, TP5448, was thermotolerant and grew at a hotter temperature (35 °C), whereas no growth was observed for isolate PLM300. This lack of growth at 35 °C was likely due to increased sensitivity to high-temperature stress, as isolate PLM300 resumed growth when shifted to a favorable temperature.

### 3.4. Mating-Type Locus and Mating Assay

Amplification of MAT1-1-1 sequences was observed in 12 isolates, whereas five isolates failed to yield a product (Figure S2). The length of the MAT1-1-1 amplicon was uniform across the 12 isolates and matched the expected size of 1 kb. No amplification was observed for the five isolates with the MAT1-2-1 primers either and were labeled as undefined for the mating-type locus. A lack of amplification due to poor DNA quality was ruled out for the four isolates, as amplicons for the ITS region were obtained using the ITS1/ITS4 primer set.

The two representative isolates, TP5448 (*T. ethacetica*) and PLM300 (*Thielaviopsis* sp.), both carry the MAT1-1-1 allele (Figure S2). These two isolates were individually paired with the five isolates negative for the MAT1-1-1 allele, as well as two MAT1-1-1-positive isolates. However, after repeated attempts, ascomata were not observed on the woody substrate placed in the media, nor were ellipsoidal ascospores visible when the spore suspension was examined.

### 3.5. Pathogenicity Test

**Figure 6:**
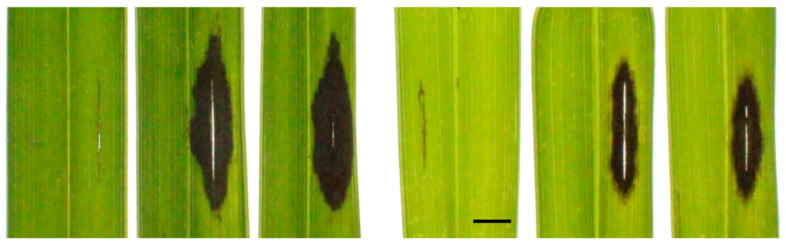
Pathogenicity assay on whole plants for *Thielaviopsis ethacetica* isolate TP5448 and *Thielaviopsis* sp. isolate PLM300. Leaflets on five-year-old *Cocothrinax guantanamensis* palms were wounded and inoculated with agar plugs carrying the inoculum. Larger necrotic lesions were observed for TP5448 three days post inoculation as compared to PLM300. Scale bar = 5 mm.

The pathogenicity of the isolates was established on whole plants as well as the berries of the palm species *Cocothrinax* and *Chamaedorea*, respectively. Lesion development on the fronds of whole palms was observed when agar plugs colonized with the fungal inoculum were placed on wound sites on the leaflets (Figure 6 and Figure S3). Inoculation without wounding failed to produce any disease symptoms. The *T. ethacetica* isolate TP5448 consistently produced lesions, with an average lesion area of 90.15 ± 6.19 mm^2^, that were larger in size compared to lesions caused by the *Thielaviopsis* sp. isolate PLM300, having an average lesion area of 45.11 ± 3.75 mm^2^ (Figure 6 and Figure S3). Koch’s postulates were completed by re-isolating the *Thielaviopsis* spp. from inoculated leaflets, and species identity was confirmed based on colony and spore morphology, whereas no lesions were observed on the control leaflets. Disease development was also observed on mature berries that were inoculated using a spore suspension carrying endoconidia and aleurioconidia, both with and without wounding. 

### 3.6. Fungicide Sensitivity

**Figure 7:**
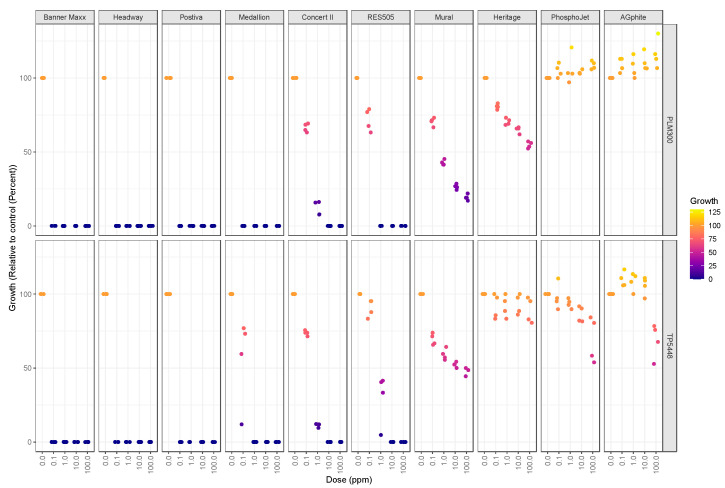
Relative growth of *Thielaviopsis ethacetica* isolate TP5448 and *Thielaviopsis* sp. isolate PLM300 in poison plate assays. Mycelial growth inhibition of the two isolates was measured in response to four doses of ten fungicides.

The mycelial growth response of the two species to ten fungicides was measured using in vitro poison plate assays. Fungicides that matched at least two of the necessary criteria, i.e., broad spectrum, systemic, injection/drench application, and labeled for ornamental use, were chosen for this study. These fungicides belong to five FRAC groups, including demethylation inhibitors (DMIs; group 3), carboxamides (SDHIs; group 7), strobilurins (QoIs; group 11), phenylpyrroles (PPs; group 12), and phosphonates (group P07) (Table 3). Three fungicides, Banner Maxx II, Headway, and Postiva, were very effective in suppressing the mycelial growth of both species, as growth inhibition was observed at the lowest concentration (0.1 μg/mL) tested (Figure 7 and Figures S4–S6). Conversely, three fungicides, Heritage WG, AGphite 57, and Phospho-Jet, showed the least growth suppression, even at the highest dose (100 μg/mL) tested (Figure 7 and Figures S4–S6). The growth of the two species responded differently to three fungicides, Medallion, Mural WG, and RES505, with all three fungicides showing better control of *Thielaviopsis* sp. isolate PLM300 as compared to *T. ethacetica* isolate TP5448 (Figure 7 and Figures S4–S6). This was also evident from the lower EC50 values for PLM300 as compared to TP5448 (Table 4). The growth inhibition in response to Concert II was almost identical for the two species.

## 4. Discussion

Characterization of the *Thielaviopsis paradoxa* isolates collected from diseased palms in Florida revealed the presence of a species complex with at least two species, *T. ethacetica* and an undescribed *Thielaviopsis* species. Multi-locus phylogeny clustered the majority of the isolates with *T. ethacetica*, whereas two isolates formed a distinct clade. Isolates from the two species showed distinct morphology, growth characteristics, and virulence, and responded differently to temperature and fungicides. The existence of multiple described and undescribed species has been previously reported in the *T. paradoxa* species complex from around the world [5,10,30], but this is the first report for distinct *Thielaviopsis* species that are pathogenic on palms in the US.

The taxonomic resolution of fungal species based solely on morphology can sometimes be misleading, as ecologically and phylogenetically distinct species may be grouped into the same genus. In such cases, improved taxon sampling and availability of DNA sequence data has helped to revise phylogenetic relationships and redefine species complexes across a diverse array of fungal genera. This was also the case for the aggregate genus *Ceratocystis* sensu lato that was amended, and seven lineages were proposed, including redefining the *Thielaviopsis paradoxa* species complex as a lineage [4]. A hallmark morphological feature of the genus *Ceratocystis*, ascomata with round bases and long necks, was not observed in the current study, as repeated attempts at mating were unsuccessful. One possible explanation is that the five isolates with undefined mating types may still have the MAT1-1 idiomorph but carry a truncated copy of the MAT1-1-1 gene. Unequal recombination resulting in partial deletion of the MAT1-1-1 gene was previously reported in the Ophiostomatales [31].

Understanding the diversity and adaptive advantages of pathogen species would offer insights to develop better disease management strategies. The two *Thielaviopsis* species characterized in this study differ in their thermotolerance as well as their aggressiveness on palms. Thermotolerance in fungi is a complex but malleable genetic trait, as evident from the routine use of adaptive lab evolution in yeast and other fungi to select strains that can grow at high temperatures for commercial, industrial, and biotechnological purposes [32]. As climate change starts to impact host and pathogen niches in the future, the two *Thielaviopsis* species are likely to contribute dissimilarly to disease pressure in palms. Global warming due to climate change is expected to alter species distribution, lead to the emergence of new pathogens and increased epidemics, and impact host–pathogen interactions [33]. A hypothesis that thermotolerant fungal species may have a selective advantage as temperatures increase was proposed as a potential mechanism for the increased prevalence of fungal diseases in mammals [34]. Subsequently, the role of thermotolerance in the emergence and spread of virulent lineages was observed in the wheat stripe rust pathogen, *Puccinia striiformis* f. sp. *tritici* [35,36].

Disease management options for Thielaviopsis trunk rot in palms are sparse. Preventative measures that discourage wounding and over-pruning and post-disease sanitation methods that reduce inoculum load are two practical approaches currently available to stakeholders [2]. Several chemical fungicides that were found to be effective in limiting mycelial growth in vitro would need additional evaluation in greenhouse and landscape trials before becoming a part of the toolbox. Other fungicides that showed hormetic effects, i.e., the promotion of mycelial growth at lower fungicide doses, would likely be excluded from further testing, as these may contribute to increased disease severity in the field [37]. Understanding the inter-species variation in fungicide responses relative to the existing pathogen species diversity is key to building a good disease management program for palms. This initial study underscores the need for targeted surveys across the range of palm- and sugarcane-growing regions to help uncover the existing diversity of *Thielaviopsis* species in the US and build better pathogen surveillance and disease diagnostics capabilities.

## Figures and Tables

**Table 1:** Location, host, and accession numbers for seventeen *Thielaviopsis* isolates characterized in this study.

Isolate ^1^	Species Name	Palm Host	Location (County) ^2^	Year	ITS	β-Tubulin	TEF1-α
PLM40	*Thielaviopsis ethacetica*	*Cocos nucifera*	West Palm Beach	2005	OR137915	OR136943	OR147317
PLM41	*T. ethacetica*	*C. nucifera*	West Palm Beach	2005	OR137916	OR136944	OR147324
PLM42	*T. ethacetica*	*C. nucifera*	West Palm Beach	2005	OR137917	OR136945	OR147320
PLM70	*T. ethacetica*	*Syagrus romanzoffiana*	Orange	2005	OR137918	OR136946	OR147325
PLM300	*Thielaviopsis* sp.	*Phoenix dactylifera*	West Palm Beach	2007	OR137919	OR136947	OR147331
PLM301	*Thielaviopsis* sp.	*P. dactylifera*	West Palm Beach	2007	OR137920	OR136948	OR147332
PLM636	*T. ethacetica*	*Wodyetia bifurcata*	Unknown, FL	2012	OR137921	OR136949	OR147321
PLM694	*T. ethacetica*	*P. dactylifera*	Manatee	2013	OR137922	OR136950	OR147326
PLM695	*T. ethacetica*	*P. dactylifera*	Manatee	2013	OR137923	OR136951	OR147318
PLM822	*T. ethacetica*	*P. dactylifera*	Miami-Dade	2015	OR137924	OR136952	OR147327
PLM823	*T. ethacetica*	*P. dactylifera*	Miami-Dade	2015	OR137925	OR136953	OR147328
PLM873	*T. ethacetica*	*C. nucifera*	Martin	2016	OR137926	OR136954	-
PLM874	*T. ethacetica*	*C. nucifera*	Martin	2016	OR137927	OR136955	OR147322
TP5448	*T. ethacetica*	*C. nucifera*	Lee	2008	OR137928	OR136956	OR147319
TPDP1	*T. ethacetica*	*P. dactylifera*	Unknown, FL	2022	OR137929	OR136957	OR147323
TPDP2	*T. ethacetica*	*P. dactylifera*	Unknown, FL	2022	OR137930	OR136958	OR147329
TPYS	*T. ethacetica*	*P. dactylifera*	Unknown, FL	2022	OR137931	OR136959	OR147330

^1^ The *T. ethacetica* isolate PLM873 was not included in the multi-locus phylogenetic analysis, as TEF1-α could not be amplified. ^2^ Name of the county in Florida from where the sample was collected.

**Table 2:** Primer pairs and PCR conditions used to generate amplicons for phylogenetic analysis and mating-type loci.

Region	Primer	Sequence (5′—3′)	Reference	PCR Cycling Conditions ^1^
ITS	ITS1	TCCGTAGGTGAACCTGCGG	White et al., 1990 [17]	95 °C 3 m; 39× [94 °C 30 s; 54 °C 30 s; 72 °C 60 s]; 72 °C 10 m
	ITS4	TCCTCCGCTTATTGATATGC		
β-tubulin	bt1a	TTCCCCCGTCTCCACTTCTTCATG	Glass and Donaldson, 1995 [18]	96 °C 2 m; 35× [94 °C 30 s; 54 °C 60 s; 72 °C 90 s]; 72 °C 10 m
	bt1b	GACGAGATCGTTCATGTTGAACTC		
TEF1-α	EF1-526F	GTCGTYGTYATYGGHCAYGT	Rehner and Buckley, 2005 [20]	94 °C 4 m; 35× [94 °C 45 s; 61 °C 45 s; 72 °C 60 s]; 72 °C 5 m
	EF1-1567R	ACHGTRCCRATACCACCRATCTT		
	EF1F	TGCGGTGGTATCGACAAGCGT	Jacobs et al., 2004 [19]	94 °C 4 m; 35× [94 °C 45 s; 60 °C 45 s; 72 °C 60 s]; 72 °C 5 m
	EF2R	AGCATGTTGTCGCCGTTGAAG		
MAT1-1	ThPara_111_F	CCACATCAGCCATTTGATTC	Wilken et al., 2018 [21]	95 °C 3 m; 35× [94 °C 30 s; 58 °C 30 s; 72 °C 60 s]; 72 °C 10 m
	ThPara_111_R	TCTCCCTGAAAAGGGTCCGT		
MAT1-2	MAT121-1F	ATACSCCAGTTCTTGTTC	This study	95 °C 3 m; 35× [94 °C 30 s; 58 °C 30 s; 72 °C 60 s]; 72 °C 10 m
	MAT121-1R	TGGGCGGTATTGATAATC		

^1^ Each PCR step is listed as a combination of two numbers: 1—temperature in degrees centigrade; 2—time, m in minutes and s in seconds.

**Table 3:** Brand name, active ingredients, FRAC groups, class, and spectrum of action for the ten fungicides used for in vitro poison plate assays.

Brand Name	Active Ingredients (Percent)	FRAC Groups	Class	Spectrum of Action
Heritage	Azoxystrobin (50)	11	Strobilurin	Broad spectrum, systemic
Banner Maxx II	Propiconazole (14.3)	3	DMIs	Broad spectrum, systemic
Concert II	Chlorothalonil (38.5); Propiconazole (2.9)	M5; 3	Multi-site; DMIs	Contact, broad spectrum, systemic
Headway G	Azoxystrobin (0.31); Propiconazole (0.75)	11; 3	Strobilurin; DMIs	Broad spectrum, systemic
Medallion WDG	Fludioxonil (50)	12	Phenylpyrrole	Contact, non-systemic
Mural	Azoxystrobin (30); Benzovindiflupyr (15)	11; 7	Strobilurin; Carboxamides	Broad spectrum, systemic
Postiva	Difenoconazole (11.5); Pydiflumetofen (6.9)	3; 7	DMIs; Carboxamides	Broad spectrum, systemic
RES505	Confidential	NA	NA	NA
AGphite	Mono- and di-potassium salts of phosphorous acid (56)	P07	Phosphonates	Systemic
Phospho-jet	Mono- and di-potassium salts of phosphorous acid (45.8)	P07	Phosphonates	Systemic

**Table 4:** EC50 values for fungicides tested against the two *Thielaviopsis* species.

Fungicide	EC50 Estimate ^1^	
	PLM300	TP5448
Banner Maxx	<0.1	<0.1
Headway	<0.1	<0.1
Postiva	<0.1	<0.1
Medallion	<0.1	0.106
Concert ll	0.182	0.217
RES505	0.129	0.543
Mural	0.266	Not estimated
Heritage	Not estimated	Not estimated
PhosphoJet	Not estimated	Not estimated
AGphite	Not estimated	Not estimated

^1^ The EC50 value was listed as ‘not estimated’ for isolates where growth inhibition was less than 50% even at the highest dose tested. The EC50 value was listed as ‘<0.1’ when no fungal growth was observed even at the lowest dose tested.

## Data Availability

All data generated or analyzed during this study are included in this article.

## Supplementary Materials

The following supporting information can be downloaded at https://www.mdpi.com/article/10.3390/jof10040247/s1: Figure S1: Phylogenetic relationship based on maximum likelihood (ML) analysis using the tef1-α gene. Sequence for *C. paradoxa* s. lat. 3 isolate C 1481 [11] was included along with previously published sequences from other *Thielaviopsis* species [6]. The branch labels represent percent bootstrap support. *Davidsoniella virescens* was used as an outgroup. Figure S2: PCR products using the MAT1-1 primers [21] from 16 *Thielaviopsis* isolates. Isolate 41A is not shown here. Figure S3: Scanned image of *Cocothrinax guantanamensis* leaflets taken from whole plants that were wounded and inoculated with agar plugs carrying *Thielaviopsis ethacetica* isolate TP5448 and *Thielaviopsis* sp. isolate PLM300. Larger necrotic lesions were observed for TP5448 (labeled as 5W) three days post inoculation as compared to PLM300 (labeled as 300W). The control uninoculated leaflets are labeled as C and 300NW. Figure S4: Mycelial growth of *Thielaviopsis ethacetica* isolate TP5448 and *Thielaviopsis* sp. isolate PLM300 in response to four doses of ten fungicides. Figure S5: Percent growth inhibition plot for *Thielaviopsis ethacetica* isolate TP5448 and *Thielaviopsis* sp. isolate PLM300. Figure S6: Growth of *Thielaviopsis ethacetica* isolate TP5448 and *Thielaviopsis* sp. isolate PLM300 in poison plate assays. Mycelial growth inhibition of the two isolates was measured in response to four doses of ten fungicides.
