# Supplementary figures and images for: Characterizing the Palm Pathogenic Thielaviopsis Species from Florida
Source: J Fungi (Basel). 2024 Mar 26;10(4):247. doi: 10.3390/jof10040247 (PMC11051176; doi:10.3390/jof10040247)

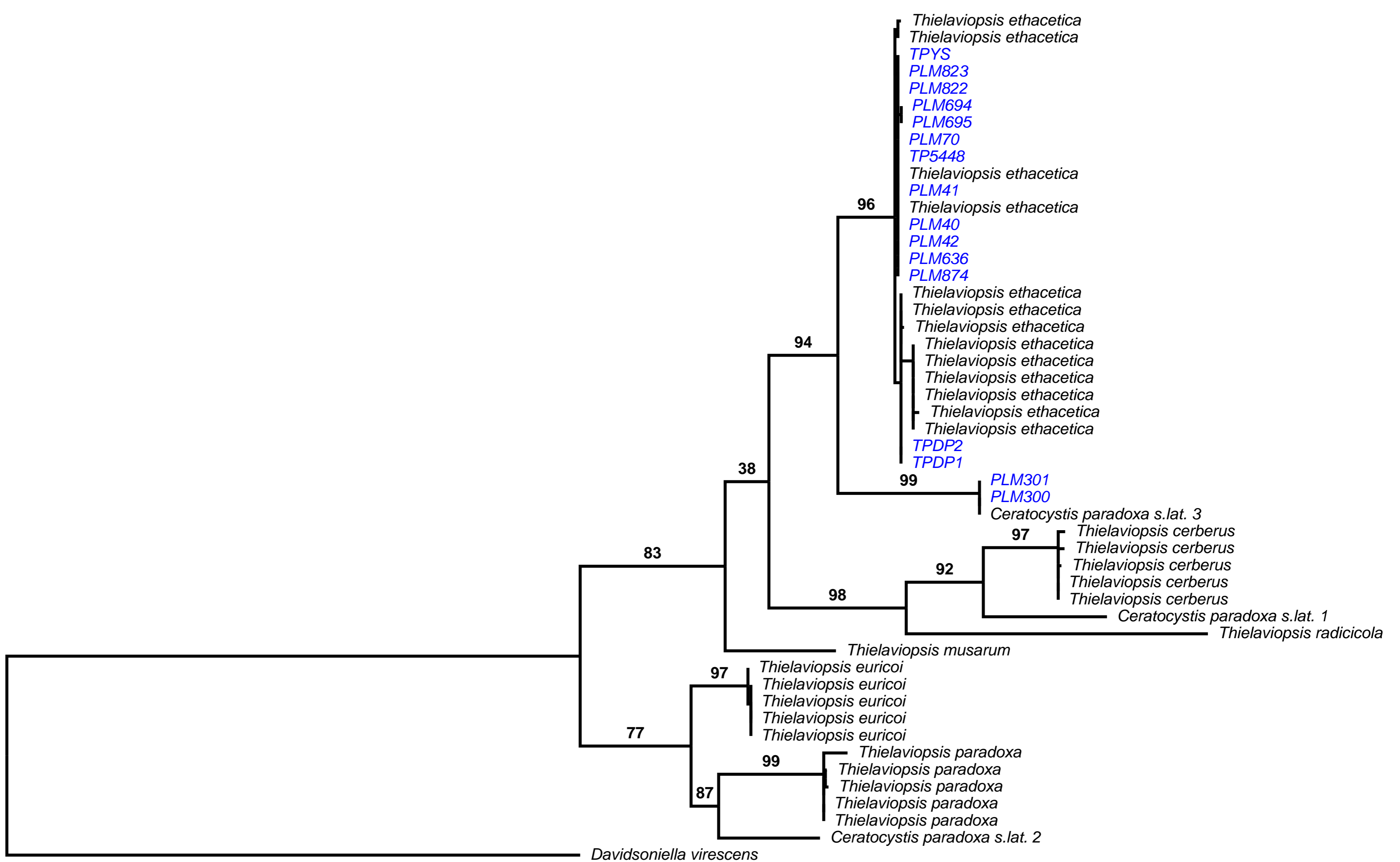

Supplement: Supplementary file 1 [file jof-10-00247-s001.zip › Figure S1_tef_16_mbenoun_c1481_with_outgroup_mafft_phy_phyml_tree.txt.pdf]

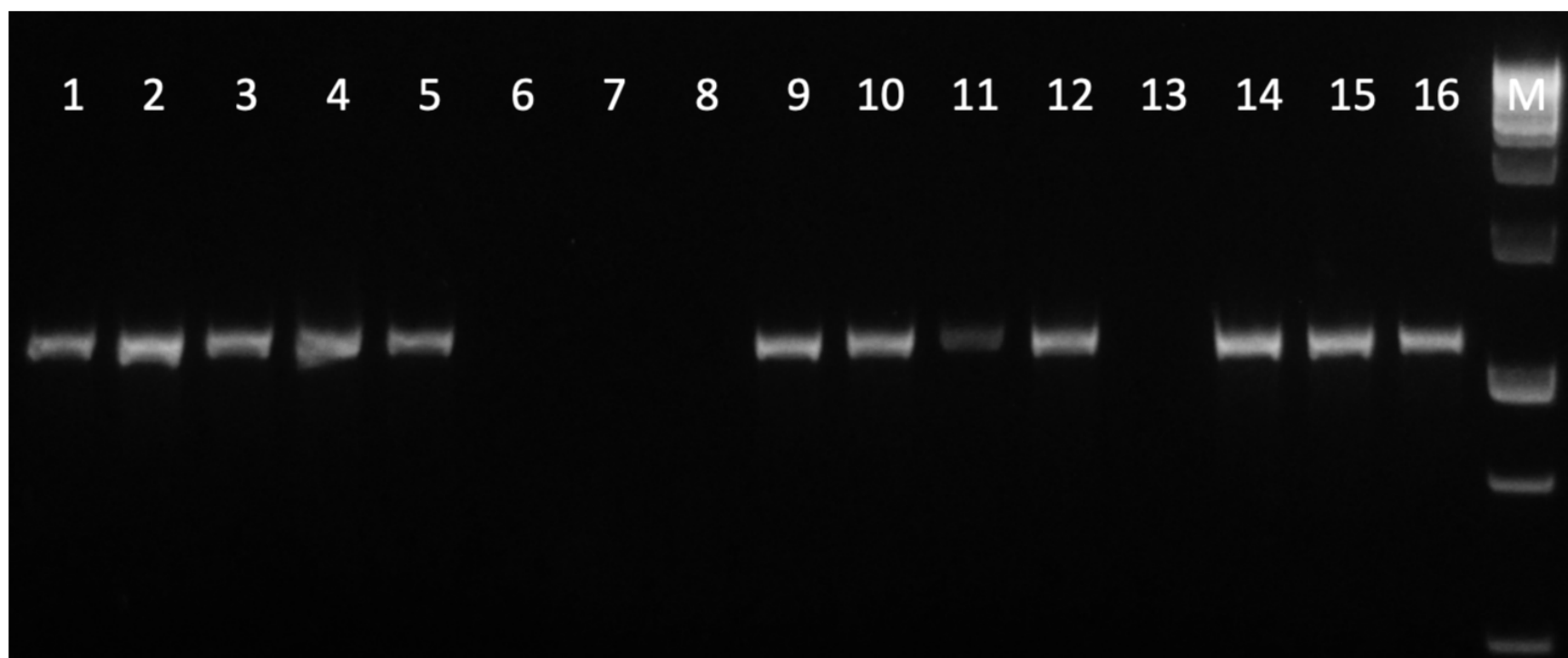

1kb

1. 40A
2. 42A
3. 70A
4. 300A
5. 301A
6. 636A
7. 694A
8. 695A
9. 822A
10. 823A
11. 873A
12. 874A
13. YS
14. TPDP1
15. TPDP2
16. 5448A

Supplement: Supplementary file 1 [file jof-10-00247-s001.zip › Figure S2_MAT.pdf]

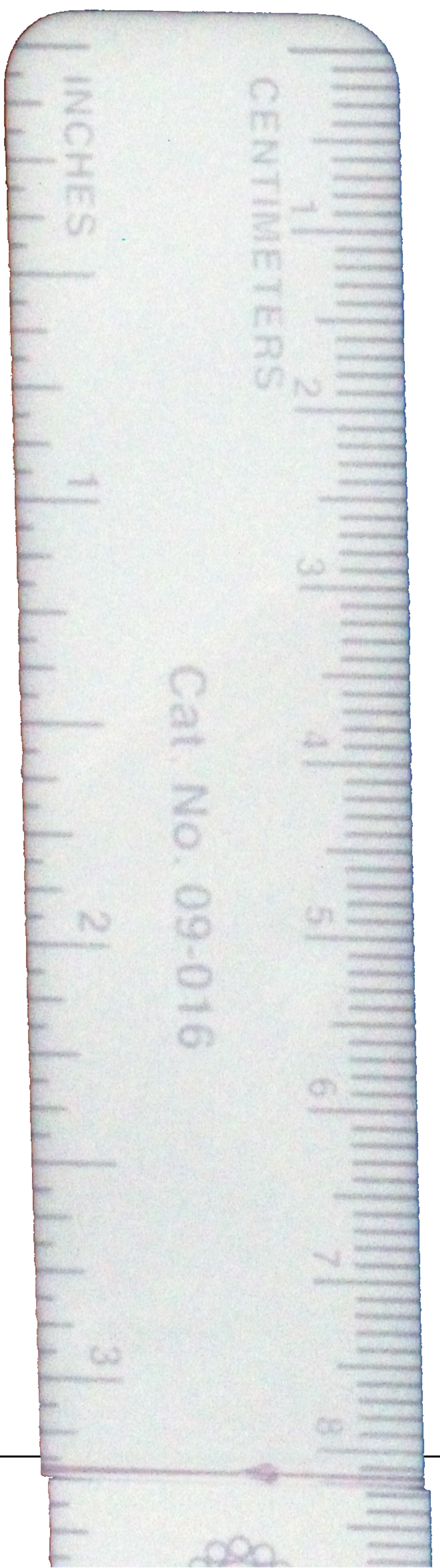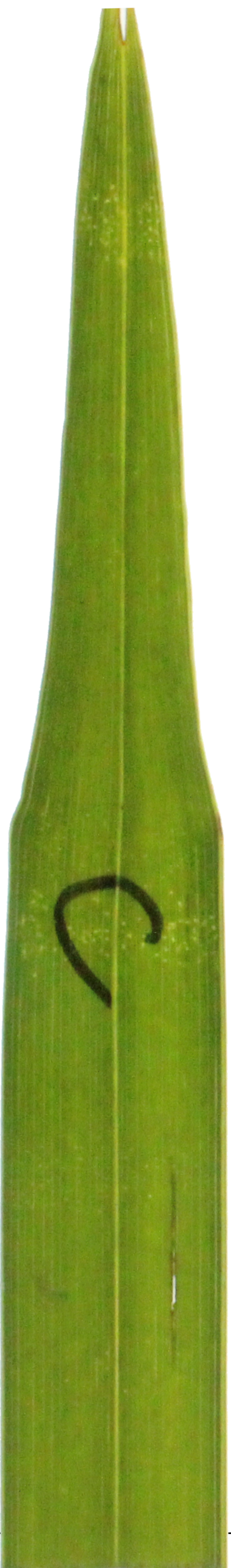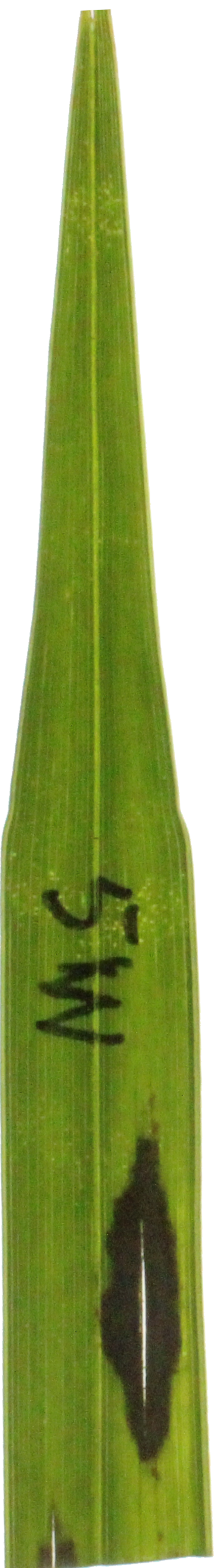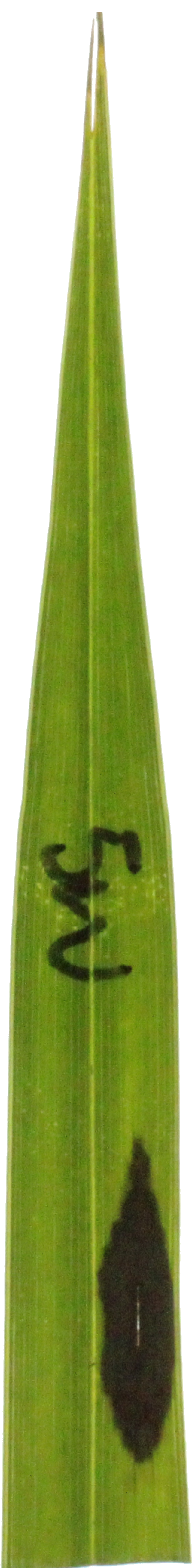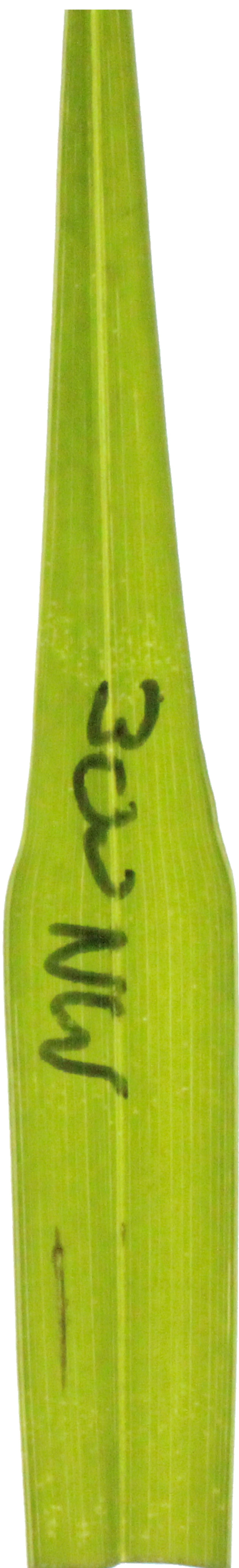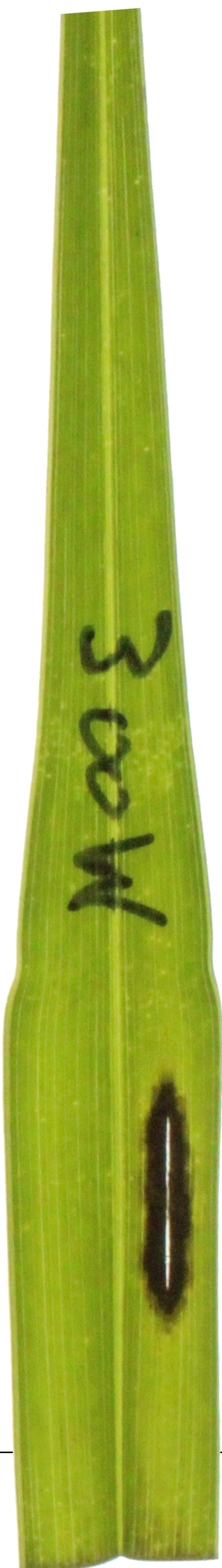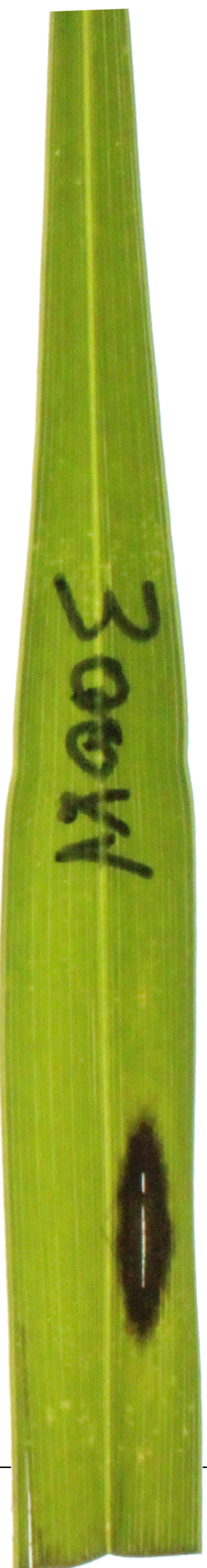

Supplement: Supplementary file 1 [file jof-10-00247-s001.zip › Figure S3_inoculation.pdf]

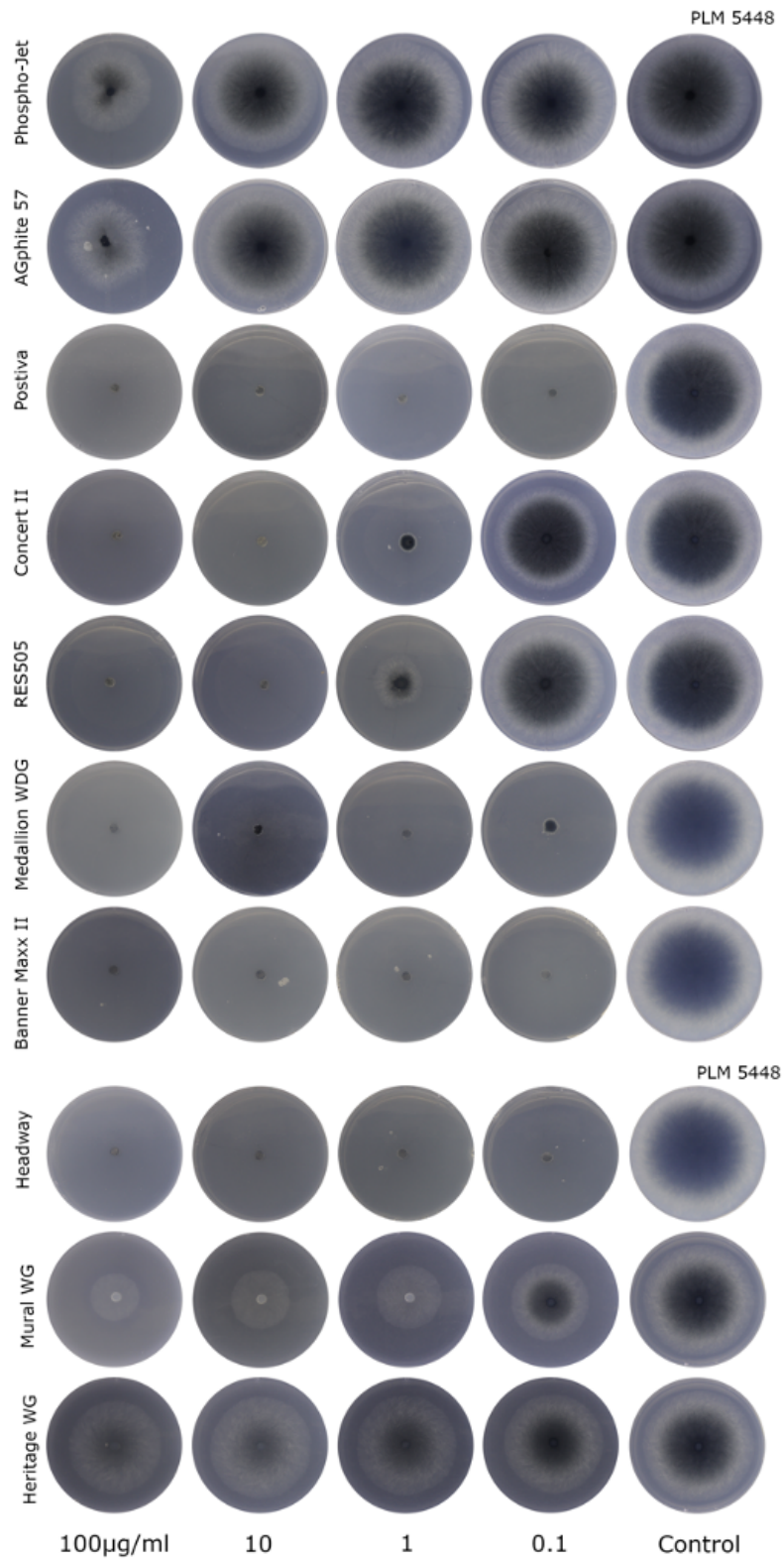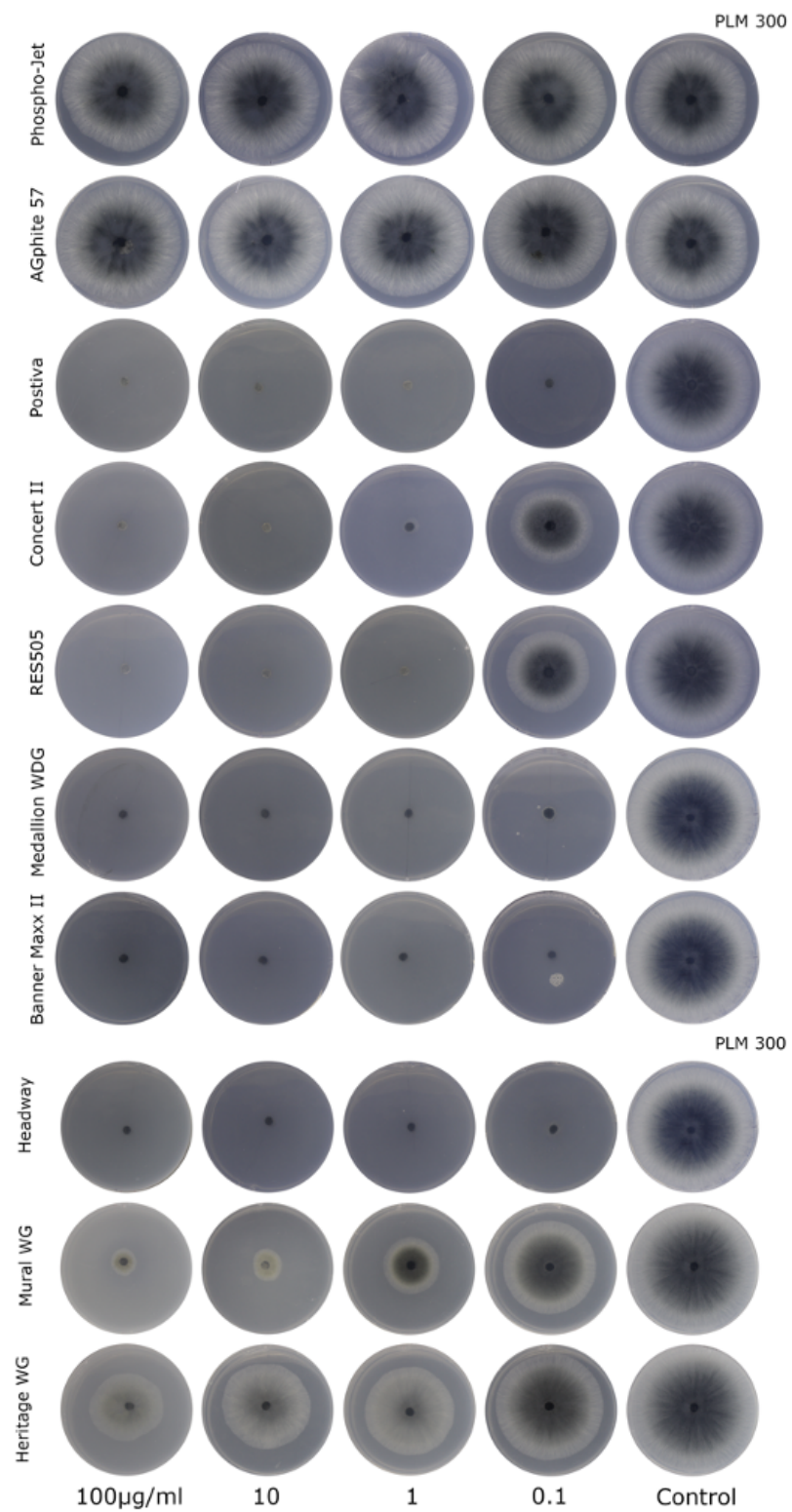

Supplement: Supplementary file 1 [file jof-10-00247-s001.zip › Figure S4_inhibition.pdf]

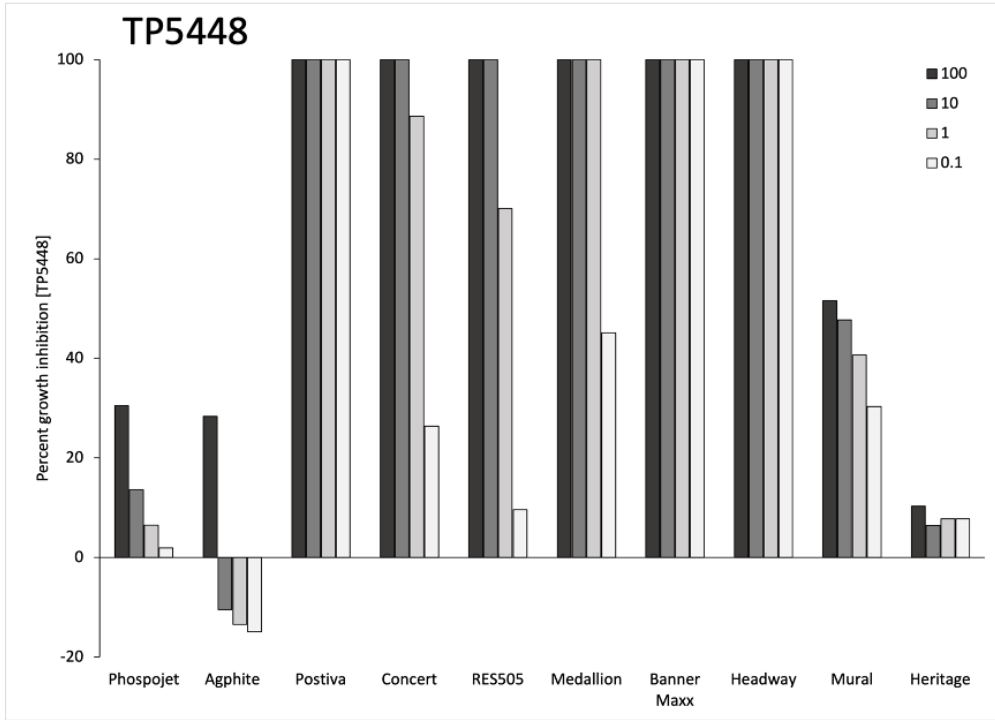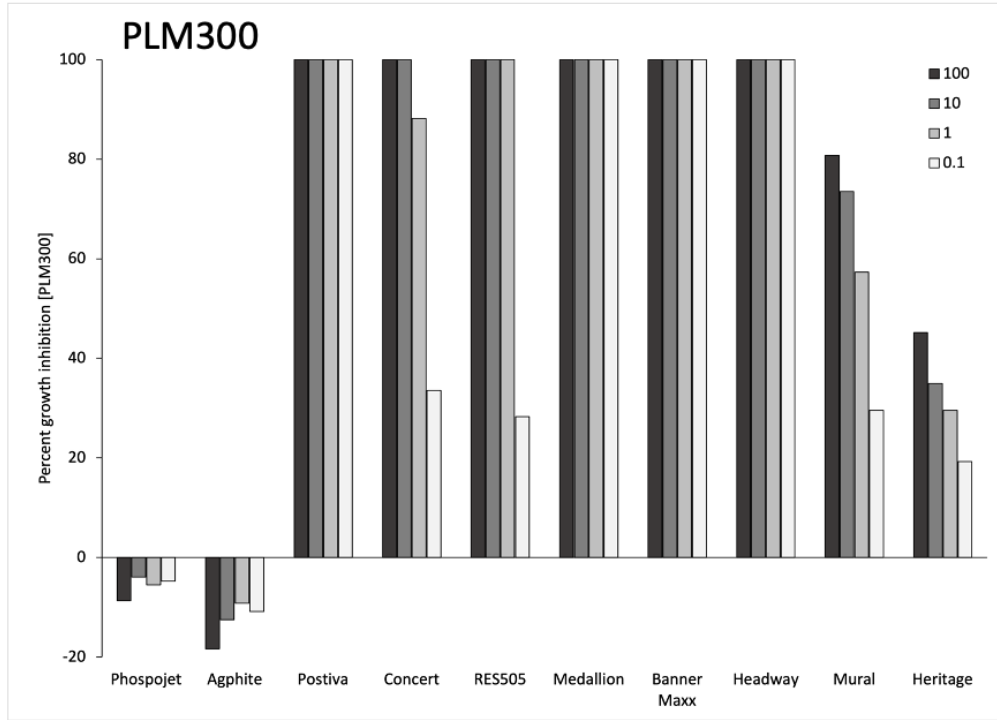

Supplement: Supplementary file 1 [file jof-10-00247-s001.zip › Figure S5_PGI.pdf]

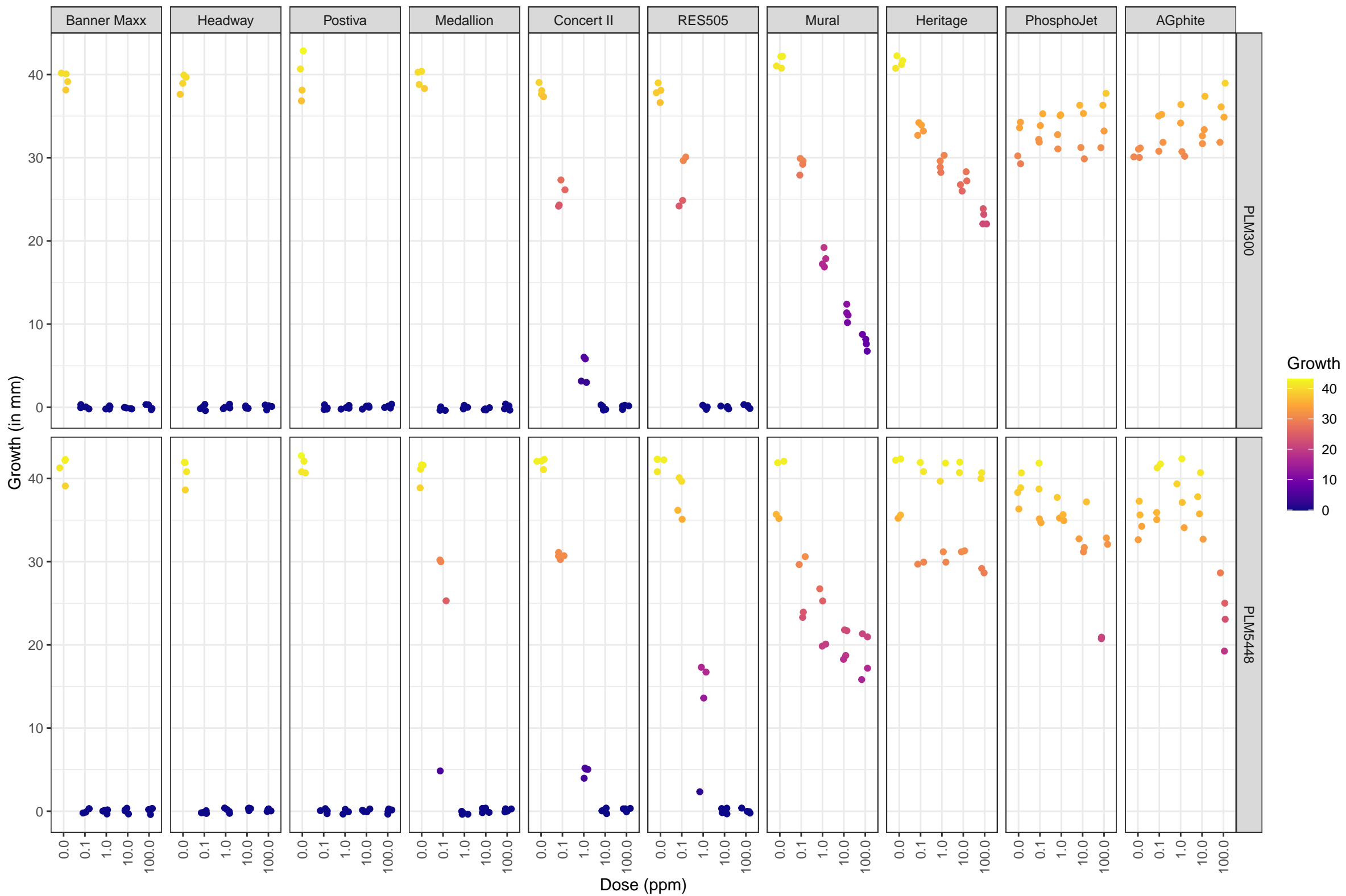

Supplement: Supplementary file 1 [file jof-10-00247-s001.zip › Figure S6_growth.pdf]
